# Editorial: Impact of the innate and adaptive immune system in driving type 1 inflammatory skin disease

**DOI:** 10.3389/fimmu.2025.1568773

**Published:** 2025-02-12

**Authors:** Mark Mellett, Judit Danis, Barbara Meier-Schiesser

**Affiliations:** ^1^ Department of Dermatology, University Hospital Zürich, University of Zürich (UZH), Zürich, Switzerland; ^2^ Department of Immunology, University of Szeged, Szeged, Hungary

**Keywords:** psoriasis, vitiligo, type 1 inflammatory skin disease, tissue-resident memory T cells (TRM cells), acne (acne vulgaris), atopic dermatitis (AD), juvenile dermatomyositis (JDM), Th1 cells

The last 20 years have witnessed a revolutionary change in how inflammatory skin diseases are treated. Immune profiling studies between lesional and non-lesional or healthy skin have provided crucial insights into the immune cell populations and culprit cytokines responsible for driving disease persistence or recurrence. These approaches have been bolstered by *in vivo* or *ex vivo* models, where cytokine neutralisation has been used to study the role of specific cytokine blockade on disease severity. Validation of the CD4+T helper (Th) subsets involved in the pathogenesis of skin disease has revealed numerous therapeutic targets such as polarising, maintenance and effector cytokines of T cell subsets. In particular, Th17 and Th2-associated cytokines, which include IL-17, IL-23, IL-22, IL-4 and IL-13 have been the target of intense pharmaceutical scrutiny and biologics targeting these cytokines have proven efficacious.

Type 1 inflammation associated with Th1 cells and Natural Killer cells, is primarily driven by TNFα and IFNγ. These protect against intracellular pathogens and tumour cells but aberrant activation is associated with a myriad of inflammatory skin conditions. While these diseases typically display a type-1 skewed immune bias, this Research Topic of two original research articles, one brief research report, five reviews and one mini-review underscores the role of both innate and adaptive immune cells in shaping the inflammatory milieu associated with type-1 inflammatory skin diseases. From acne, to eczema, psoriasis and vitiligo, a consistent theme emerges: these diseases are multifactorial processes with a dynamic interplay of multiple cellular and molecular players.

Yang et al. provide a comprehensive and timely review of rosacea that exemplifies this complexity of type 1 skin disease. They examine the molecular interactions that drive rosacea pathogenesis, in particular the role of LL37, the human Cathelicidin peptide that displays a wide range of immunomodulatory functions. In rosacea, LL37 exerts pleiotropic influence on effector cells - inducing the release of IL-8 from keratinocytes, VEGF from endothelial cells (mediated by mTORC1) and type I Interferons from plasmacytoid dendritic cells (pDCs). IL-8 serves as a chemoattractant for neutrophils and VEGF, in addition to promoting angiogenesis, stimulating Th1 cell differentiation. Through activation of the transient receptor potential vanilloid 4 (TRPV4) LL37 activates macrophages and mast cells.

Kallikrein 5 (KLK5) cleaves cathelicidin to the active LL37 peptide after Toll-like receptor 2 (TLR2) activation and this TLR2-KLK5-LL37-mTOR axis is a main therapeutic strategy for disease management. Additionally, new treatment strategies, such as targeting Th1/Th17 cells, the JAK/STAT pathway or the use of VEGF inhibitors to curtail angiogenesis are highlighted.

The brief research report from Raupov et al. provides insight into the role of type I interferon (IFN-I) signalling in juvenile dermatomyositis. Juvenile dermatomyositis is an idiopathic inflammatory myopathy characterised by muscle weakness and eczema. Raupov et al. show a significant correlation between elevated IFN-I scores and skin disease activity, highlighting the potential of serum IFN-I as a promising biomarker for skin involvement but also arthritis in these patients.

Two comprehensive reviews by Jin et al. and Huang et al. provide an in-depth look at the complexity of acne vulgaris, challenging the traditional perception of this condition as a mere superficial skin issue.

Jin et al. delve into the immune processes involved in the pathogenesis of acne vulgaris, detailing the role of microbiome dysbiosis and the innate and adaptive immune response to *Cutibacterium acnes* (*C. acnes*), *Staphylococcus* and *Malassezia*. Peptidoglycan, lipoteichoic acid and short-chain fatty acids of *C. acnes* induce pattern-recognition receptor activation on keratinocytes, sebocytes and monocytes, which release anti-microbial peptides and cytokines that tailor the adaptive immune response. Additionally, Jin et al. describe the role of neuropeptides, such as Corticotropin-releasing hormone and Substance P. Huang et al. build on this by detailing the differential cellular responses at different stages of disease development. Th1 and Th17 cells play an important early role in microcomedones when follicles rupture and neutrophils are attracted in large numbers to further drive inflammation. Interestingly, mast cells also play a role in early acne lesions, being recruited by keratinocyte-produced stem cell factor, and are a source of IL-17A in acne lesions.

These reviews underscore the need to study the temporal and spatial dynamics of immune activity in acne development, to facilitate more targeted treatments.

In an original article, Seiringer et al. use spatial transcriptomics to uncover the active role of sebaceous glands in psoriasis vulgaris and the pathogenesis of atopic dermatitis. Both diseases show altered lipid metabolism in the sebaceous gland transcriptome compared to non-lesional sebaceous glands and the upregulation of inflammatory mediators including serum amyloid A1. In atopic dermatitis, a number of genes associated with lipid skin barrier formation have been identified. Interestingly, genes such as *ALOX15B*, an important regulator of fatty acid metabolism, and *CCL17*, two of the spatially variable genes upregulated here are known to be induced by type-2 cytokines, IL-4 and IL-13 in macrophages, suggesting a potential role of the sebaceous gland in atopic dermatitis. In psoriatic tissue, sebaceous gland gene expression profiles showed an increase in type I interferon and anti-microbial peptide expression but also heightened differentiation and SUMOylation. These data present sebaceous glands as immunomodulatory structures that contribute to the shaping of the immune environment in skin disease.

Morelli et al. investigate the difference between anti-PD-1-induced psoriasis in three oncology patients with samples of chronic plaque psoriasis and paradoxical psoriasis (resulting from anti-TNFα treatment). Their original research article shows that this immune-related cutaneous adverse event is immunologically similar to plaque psoriasis. Conversely, the innate immune arm, i.e. the type I interferon response and myeloid cell involvement, plays a lesser role in anti-PD-1-induced psoriasis compared to paradoxical psoriasis. Interestingly, next-generation sequencing showed that all three patients harboured SNPs associated with an increased risk of psoriasis, including specific ERAP1 haplotypes that may be involved in the generation of certain autoantigens for HLA-class I presentation and autoimmune CD8+ T-cell activation. All three patients displayed enhanced expression of the psoriasis autoantigen ADAMTSL5, which is also found in melanoma tissue. The authors suggest that ADAMTSL5-specific T-cell responses that protect against the tumour may trigger the onset of psoriasis in these patients.

Liu G. et al. probe into the double-edged role of tissue-resident memory T (Trm) cells. Acting as sentinels, Trm cells remain in peripheral skin tissue for extended periods. The review discusses how CD4+ and CD8+ Trm cells play both protective and pathogenic roles. CD4+ Trm cells can circumvent the innate immune response and induce effector functions upon antigen recall. TGFβ, TNFα, IL-33 and IFNγ maintain CD103 and CD69 expression on CD4+ Trm cells, facilitating their retention in tissues, while IL-15 plays this role in CD8+ Trm cells. In psoriasis and vitiligo, CD4+ Trm and CD8+ Trm cells play a pathogenic role in disease recurrence, while in melanoma these CD8+ Trm cells actively hinder tumour progression and enhance immunotherapy responses.

Liu S. et al. highlight the multifaceted role of macrophages in numerous type-1 inflammatory skin diseases and also atopic dermatitis, melanoma and cutaneous T-cell lymphoma. In psoriasis, macrophages can express two important autoantigens, LL37 and ADAMTSL5 and produce important chemokines, including MIP-1α and –β. In atopic dermatitis, M2-like macrophages are a major source of IL-31, a main trigger of pruritus. M2-like macrophages also play a role in Bullous pemphigoid (BP), where these cells produce CCL18. Mouse studies show that macrophages may drive the blistering associated with BP. In melanoma, macrophages have been implicated in promoting metastases and angiogenesis via HIF-1α and HIF-2α. The plasticity of macrophages to adapt to different inflammatory environments underscores their importance in immune responses and potential therapeutic targeting.

Migayron et al. offer a compelling perspective on the role of type-2 immunity in type-1-associated skin diseases, vitiligo, localised scleroderma and alopecia areata. Their mini-review describes the complex role of Th2 cytokines and chemokines, IL-4, IL-13 and TSLP in these diseases and the cross-talk between mast cells and CD8+ T cells. Whether type-2 inflammation contributes to pathology or is a protective mechanism remains to be fully clarified in clinical subsets, which will reveal how to better stratify patients for therapeutic intervention.

To summarise, this Research Topic highlights new insights into our understanding of how we classify and characterise type 1 skin diseases. In particular it illuminates the rich but detrimental, immune cross-talk that drives disease persistence. We believe that this Research Topic will serve to inform and inspire further research in this field with the goal of identifying new therapeutic targets for a plenitude of type 1 skin diseases.

